# Colour vision deficiency and sputum colour charts in COPD patients: an exploratory mixed-method study

**DOI:** 10.1038/s41533-021-00225-z

**Published:** 2021-03-04

**Authors:** Sunita Channa, Nicola Gale, Emma Lai, Lara Hall, Mark Quinn, Alice M. Turner

**Affiliations:** 1 Institute of Applied Health Research, University of Birmingham, Birmingham, UK; 2 School of Social Policy, Health Service Management Centre, University of Birmingham, Birmingham, UK

**Keywords:** Respiratory signs and symptoms, Chronic obstructive pulmonary disease

## Abstract

Sputum colour may mark bacterial involvement in acute exacerbations of chronic obstructive pulmonary disease (COPD). However, whether colour vision deficiency (CVD) in COPD patients could impact the use of sputum colour charts as part of a guide to antibiotic use in exacerbations is unknown. This study used an exploratory mixed-method approach to establish the likelihood that COPD patients will be colour blind and whether this would result in the sputum colour chart being unusable in the context of the patients’ self-management of their condition. CVD is under-reported in primary care and comorbidities in COPD patients increase the risk of acquiring CVD. Participants diagnosed with CVD and risk of acquiring CVD were able to use the sputum colour charts. Colour charts are likely to be usable even in the context of undiagnosed CVD in COPD patients.

## Introduction

Chronic obstructive pulmonary disease (COPD) is a long-term respiratory condition, currently the fourth leading cause of death globally^1–3^. Acute exacerbations of COPD (AECOPD) are acute events leading to the worsening of symptoms from the patient’s usual stable state^4^. Research suggests that early intervention strategies for AECOPD, including self-management packages, can reduce disease progression, hospital admissions and improve quality of life^5–8^.

Usual care in the United Kingdom (UK) includes a self-management plan and a ‘rescue pack’ of both antibiotics and steroids, which patients are advised to take when they suspect an exacerbation^9^. Although only about half of AECOPD are caused by bacteria, antibiotics are widely prescribed^10^. As a result, patients who are prescribed rescue packs need to be educated about risks of overusing medication and benefits of seeking medical evaluation if unsure about their symptoms, as a safety net^11^.

**Fig. 1:**
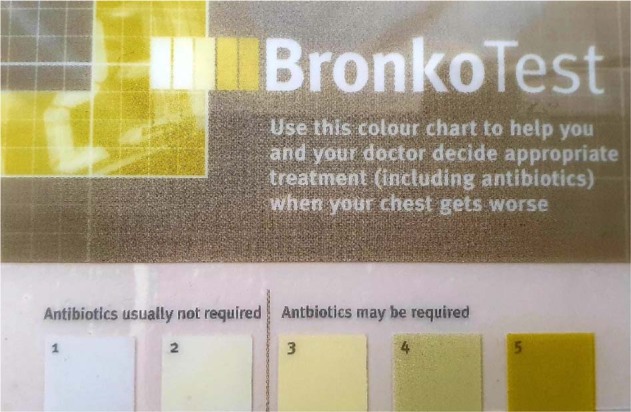
Bronkotest® card with the sputum colour chart. 1 and 2 represent white to light yellow classification of sputum colour and 3, 4 and 5 are increasingly purulent.

Sputum colour is a marker of neutrophilic inflammation and bacterial infection^12^. The Bronkotest 5-point chart is a standardised method of classifying sputum colour whereby 1–2 are white to light yellow and 3, 4 and 5 are increasingly purulent (Fig. 1)—greener colours indicate the presence of bacterial infection. In an AECOPD population, the presence of green sputum has a positive predictive value for bacterial infection of 80%, and the absence of green sputum has a negative predictive value of 93%. This suggests sputum colour is a tool with the potential to reduce inappropriate antibiotic use with low risk of harm.

Although colour charts may help patient’s self-manage antibiotics, issues of usability could arise with patients who have colour vision deficiency (CVD). CVD may be congenital or acquired^13^ and affects ~8% of men and 0.5% of women globally. People acquire CVD due to age-related macular degeneration, smoking or diseases such as diabetes mellitus and glaucoma^14^. CVD can be separated into three different types—red-green (protanopia or deuteranopia) or blue-yellow (tritanopia). Although there is evidence to suggest that COPD may cause considerable retinal and optic nerve damage^15^, and heavy smoking has been associated with losses in red-green colour vision^16^, little is known on how CVD impacts COPD patients.

Effectiveness of the Bronkotest colour chart in self-management of AECOPD is currently being evaluated in a randomised controlled clinical trial (Colour COPD; ISRCTN 14956629). This sub-mixed methods substudy aimed to investigate its utility by establishing the prevalence of CVD in primary care, and the usability of the chart in people with diagnosed and undiagnosed CVD.

## Results

### Prevalence of diagnosed CVD and risk factors for acquisition of CVD

A total of 295,879 records were extracted from The Health Improvement Network (THIN) database, an anonymised database of electronic UK primary care records from ~6% of the population^17^. The prevalence of CVD was low and mainly in men (Table 1), with variation in reporting by age: 0–6 (5.06%),17–30 (36.71%), 31–40 (30.38%) and 61–70 (3.8%).

**Table 1:** Demographic characteristics of patients diagnosed with CVD from the THIN Database.

Demographic feature	*n* = 79 CVD participants, median (IQR) or %
Age (years)	32 (15)
Male	78 (98.7%)
Ethnicity	
White	39 (49.4%)
Others	1 (1.3%)
Missing	39 (49.4%)
Read codes	
F485.11 (colour blind)	73 (92.4%)
F485100 deutan defects	1 (1.3%)
F485z00 (colour blindness not otherwise specified)	5 (6.3%)

### Prevalence of factors predisposing COPD patients to acquired CVD

This was assessed in 593 participants in the integrated care for COPD (INTEGR COPD) trial; characteristics are shown in Table 2. On average, patients had moderate COPD and a heavy smoking history; 49% were current smokers, 37% were ex-smokers and 4% had never smoked.

**Table 2:** Characteristics of the INTEGR COPD study cohort.

Feature	Mean (SD) or %
Age	68 (11)
Pack-year history	47 (30)
Male sex	52
Prevalence of comorbidities relevant to CVD	15
FEV1 (L)	1.56 (0.64)
FEV1 % predicted	62.7 (20.5)

Comorbidities that could increase the risk of CVD were common: for example, 14% (*n* = 83) had an underlying diagnosis of diabetes.

### Prevalence of undiagnosed CVD

This was assessed by testing colour vision using the Chroma Test in patients ‘at risk’ of CVD (*n* = 13, Table 3) due to significant smoking (COPD patients, *n* = 10) or other health conditions (*n* = 3). Seven participants had CVD identified, of whom five had COPD. No correlation was seen between smoke exposure and degree of CVD (*r* < 0.38, *p* > 0.14 for protanopia and *r* < 0.25, *p* > 0.34 for tritanopia).

**Table 3:** Characteristics of the participants assessed for undiagnosed colour vision deficiency (CVD).

	Age	Sex	COPD	Smoking pack-year history	Associated medical conditions	Colour vision deficiency
	69	Male	√	34	Cataracts	No CVD
	20	Female	x	0	Stargardt’s macular dystrophy	Protanopia
	23	Female	x	0	Type 1 diabetes mellitus	Tritanopia
	78	Male	√	30	Type 1 diabetes mellitus	No CVD
	75	Female	√	26	Type 2 diabetes mellitus; cataracts	Protanopia
	72	Female	√	75	Type 2 diabetes mellitus; cataracts	No CVD
	20	Female	x	0	Type 1 diabetes mellitus	No CVD
	74	Female	√	22	Cataracts	Tritanopia
	62	Male	√	49	N/A	No CVD
	73	Male	√	90	N/A	Tritanopia
	75	Male	√	32	N/A	Tritanopia
	83	Male	√	44	Type 2 diabetes mellitus; cataracts	Protanopia and tritanopia
	64	Male	√	38	N/A	No CVD
Median (IQR)	72 (20)	—	—	32 (22)	—	—

### Usability of sputum colour chart in people with, or at risk of, CVD

This was assessed qualitatively in the 13 ‘at risk of CVD’ patients in Table 3, plus 8 patients with known CVD (Table 4). A total of eight interviews took place (four focus groups and four individual interviews).

**Table 4:** Characteristics of the participants with known colour vision deficiency (CVD).

	Age	Sex	Smoking pack-year history	Associated medical conditions	Pretest colour vision deficiency	Posttest colour vision deficiency
	81	Male	0	Bronchiectasis	Red/green	Red/green
	40	Male	20	N/A	Red/green	Red/green
	19	Male	0	N/A	Deuteranopia	Deuteranopia
	20	Male	0	N/A	Red/green	Red/green
	62	Male	0	Asthma	Red/green	Red/green
	27	Male	0	N/A	Red/green	Red/green
	51	Male	0	N/A	Red/green	Red/green
	41	Male	0	HNPP	N/A	Red/green
Median (IQR)	40.5 (19)	—	—	—	—	—

*HNPP* hereditary neuropathy with pressure palsies.

The results presented focus on the usability and perceived value of the chart: Although there were varied opinions, most known CVD participants felt able to distinguish between colours and confident about use:

there are like marked differences with the colours for me [although] I couldn’t tell you what colours they were (Pt#6)

I have no difficulty whatsoever with these colours. (Pt#1)

However, two of the participants felt that there might be some problems:

Numbers one and two are very similar. Three is slightly darker than two and then it gets easier to see the difference. (Pt#5)

So, five is definitely different. The others pretty much blend into one. (Pt#2)

The participants raised concerns about the embarrassment of having to ask another person (e.g. family member) for a second opinion on their sputum colour. They were concerned about the subjective nature of colour perception, particularly around the threshold of whether to use antibiotics. They noted the implications of this:

I would be wary of taking antibiotics unless it was necessary. Because antibiotics resistance is like a big thing. And you shouldn’t take antibiotics unless you very much need to. (Pt#3)

The majority of undiagnosed CVD participants found colours ‘easy’ to distinguish. One participant reported colour one and two (see Fig. 1) were very similar, albeit distinguishable (‘they are slightly different’). People with COPD agreed that chart colours were an accurate representation (‘very similar’) to their daily sputum.

They picked out aspects of design that were helpful, such as the line indicating the cut-off for antibiotic use:

I think the line that you have after two, um, would sort of imbed in your memory, once you get into those colours, you know what you’re going to do. (Pt#11)

However, some felt the wording ‘antibiotics may be needed’ once the colour exceeds stage 3 could be ambiguous. Most participants were happy using it without assistance apart from one who preferred reassurance from a doctor; although they would rely on their embodied experience rather than the chart alone:

But if I’m up there (points to 5) *makes a cough noise* then I know and I can feel it particularly. It’s more than just the colour. (Pt#5)

While participants generally agreed the design was practical and attractive, they suggested improvements of font size and background:

‘even with the corrective glasses I find that a bit [difficult to read]…especially the small text’ ‘The Bronkotest text is very clear. The small text above the boxes is quite small and could be darker on the light background to make it clearer and I think it could make actually the colours a bit bigger’. (Pt#7)

Known CVD participants suggested that they would prefer a white background consistent with instructions in the booklet to cough up sputum onto white tissue: ‘The card itself would have to have a white background. Because this is just off white and it doesn’t help at all’ (Pt#3).

Some participants also recommended being able to write their diagnosis on the card which would be useful in case of an emergency:

I’ve got COPD so then they have the reference and they know that you are that patient, so if you did happen to collapse you’ve got that identification at the same time. (Pt#3)

Nonetheless, participants were unanimous in seeing the chart’s value for the health system: ‘Because it saves doctor’s time as well’ (Pt#12) and in terms of its use to maintain individual health: ‘I would say I would use this card and this colour chart just to short cut your way to better health really… It’s a good warning sign’ (Pt#11). One participant summarised the sputum chart as a ‘time saving, self-help device’ (Pt#2).

## Discussion

Our study has shown that CVD is poorly recorded in the primary care record and that undiagnosed, acquired CVD has the potential to be highly prevalent in COPD patients; despite this sputum colour charts seem usable.

Congenital CVD is a common genetic disorder that is more prevalent in males (as in our data) due to the recessive X-linked disorder^14,18^. The results of our study showed a low recorded prevalence of CVD: 0.055% in males and 0.00065% in females. This is an indication that CVD recording in primary care may not be reliable, in particular, acquired CVD seems less likely to be recorded. Although CVD is untreatable, it can be argued that testing should be routine in patients who might need to visually self-manage their condition through colour detection. CVD has no structured screening^19^ and patients may not view their CVD as a condition that requires reporting to a general practitioner (GP) (and thus coding within the medical record); this is likely to explain the low prevalence in THIN.

The Chroma Test results demonstrated that 54% of participants at risk of acquiring CVD had significant undiagnosed colour vision problems, and the epidemiological data were supportive of the fact that comorbidity predisposing to CVD was common in COPD. Patients with diabetes may develop CVD^20^ and chronic cigarette smoking may lead to diffuse colour vision disturbance^16,21^ and higher colour discrimination thresholds when compared to non-smokers^16^. Thus, there is a likelihood of undiagnosed CVD in COPD populations.

The qualitative study highlighted that the majority of participants were confident in using the sputum colour chart regardless of their CVD and felt it beneficial. Other studies using the Bronkotest colour chart in non-CVD populations express similar views^22,23^.

Although CVD participants expressed uncertainty about antibiotic use due to the wording on the sputum chart, these views were not shared with COPD participants who they felt confident recognising physical symptoms to establish the need for antibiotics. Research suggests participants who are more skilled in a subject, in this case more familiar with long-term COPD management, are more confident and so are not affected by external sources of ambiguity^24^ (in this case the use of the phrase ‘may be required’).

There is evidence to suggest that patients are not currently able to correctly distinguish when they have a bacterial infection^25^, although sputum colour is part of the Anthonisen criteria for classifying exacerbation type. The sputum colour chart could help COPD patients to move closer to this classification themselves, instead of relying on their own judgement. Since CVD is likely to be under-reported in the medical record, and potentially undiagnosed even if present, direct questioning about colour vision and checking that COPD patients can distinguish colour differences on the chart would be important during education about chart use whether or not they have a formal diagnosis of CVD. Similar principles might apply to patients with a range of respiratory conditions such as asthma and bronchiectasis.

Strengths include generalisability to primary care in the UK, since THIN is a representative sample. The INTEGR COPD cohort also has this strength, since it is conducted in UK primary care, but deficiencies in coding should have been overcome by detailed baseline assessment. The main limitation is the small sample size for the colour vision testing and qualitative work. However, since the qualitative data reached saturation with this sample size, it is not likely to have markedly affected results on usability. Wider testing could provide greater reassurance about aspects of the design, given that some participants felt this could be improved. Furthermore, since CVD did not appear to affect usability it is debatable how much value widespread Chroma testing seeking undiagnosed CVD would have in COPD. Finally, this substudy cannot answer whether the chart is actually useful in self-management, although the parent trial will do so.

In conclusion, the study has demonstrated that GP records underreport CVD and that there may be high rates of undiagnosed CVD in COPD. Nevertheless, sputum colour charts remain usable.

## Methods

This mixed-method study was carried out between January 2020 and March 2020. The research was approved by the local Ethics Committee (ERN_19-1277) and written informed consent was obtained.

### Prevalence of diagnosed CVD and risk factors for acquisition of CVD in COPD

Data were sourced from THIN to determine the recorded prevalence of CVD. Within THIN, a random sample of 295,779 patients was extracted and CVD was sought using 11 read codes related to CVD via the Data Extractor for Epidemiological Research. This dataset was chosen as the primary care record may be used to determine diagnoses for patients, and thus whether a colour-based intervention is appropriate for them.

Data were also sourced from a primary care COPD cohort recruited into a trial of integrated care for COPD (INTEGR COPD: NCT03482700). Records from all patients who had completed a baseline assessment in the intervention arm (*n* = 593) were examined to ascertain the prevalence of conditions increasing the risk of developing acquired CVD. This dataset was chosen as it represents the population in whom sputum colour charts might be used in practice. INTEGR COPD was ethically approved (17/SC/0447) and all patients undergoing procedures beyond usual care gave informed consent. Descriptive statistics were used to describe the prevalence of CVD according to demographic features.

### Prevalence of undiagnosed CVD

Patients with COPD, who did not self-report CVD, and exhibited risk factors for acquiring CVD, defined as a history of heavy smoking (≥20 pack-years), macular degeneration or a neurological or ophthalmological condition known to associate with CVD, were recruited mainly from INTEGR COPD. Three people at risk of CVD due to an underlying medical condition were recruited through advertisements. All participants underwent a colour vision test called Chroma Test, which is a computer-based method of testing CVD^26,27^. Standard deviations (SDs) described how far patients were from normal vision for their age and visual acuity^28^; SD values >1.6 above the mean indicate CVD.

### Usability of colour charts

An opportunistic sample of participants with COPD assessed for undiagnosed CVD, and those with known CVD (with or without COPD) were recruited for a parallel qualitative study exploring their views on the sputum colour chart. Those with known CVD were recruited using digital and poster advertisements on the University campus and through patient groups; their CVD diagnosis was confirmed by Chroma Test.

Semistructured group or individual interviews lasting 30–60 min were conducted by two researchers using a topic guide (Table 5) exploring perceptions towards usability and accuracy of colour sputum chart. Interviews were anonymised, audio-recorded and transcribed verbatim. Transcripts were read and re-read to ensure familiarisation, and then analysed using the framework method^29^.

**Table 5:** Interview topic guide.

Interview questions
1. How would you describe the different colours on the colour chart in front of you?
2. Are there any parts of the chart where it is hard to tell the difference between the colours?
3. If you needed to do this on your own, how confident would you be in reading the chart with no assistance?
4. How much would you trust using this colour chart to be accurate, or would you want to check with a doctor or health professional?
5. What are some of the reasons why people might want to use a colour chart?
6. What are your thoughts on each of these reasons?
7. Can you anticipate any problems using it in this way?
8. What would you say is the best bit of it and the most challenging part of the chart to use?
9. Would anyone like to add anything else now?

For protocols used in the study, refer to: 10.21203/rs.3.pex-1238/v1^30^.

### Reporting summary

Further information on research design is available in the Nature Research Reporting Summary linked to this article.

## Supplementary information

Reporting Summary

## Data Availability

The data sets generated during and/or analysed during the current study are available from the corresponding author on reasonable request.
